# Fatty acid variability in three medicinal herbs of *Panax* species

**DOI:** 10.1186/1752-153X-7-12

**Published:** 2013-01-21

**Authors:** Xiao-Jing Zhang, Li-Li Huang, Xiu-Jiang Cai, Peng Li, Yi-Tao Wang, Jian-Bo Wan

**Affiliations:** 1 State Key Laboratory of Quality Research in Chinese Medicine, Institute of Chinese Medical Sciences, University of Macau, Macao, PR China; 2 Department of Obstetrics and Gynecology, Sun Yat-Sen Memorial Hospital, Guangzhou, China

**Keywords:** *Panax* species, Fatty acids, Gas chromatography–mass spectrometry, Multivariate statistical analysis, Principal component analysis, Hierarchical cluster analysis

## Abstract

**Background:**

Fatty acid profiling has been widely used in the bacteria species identification, we hypothesized that fatty acid characteristics might discriminate the *Panax* herbs according to species. To test the hypothesis, fatty acids of *Panax* species, including *Panax ginseng*, *Panax notoginseng* and *Panax quinquefolius*, were characterized and compared using gas chromatography–mass spectrometry (GC-MS) followed by multivariate statistical analysis.

**Results:**

The content of investigated 11 fatty acids, including myristic acid, pentadecanoic acid, palmitic acid, palmitoleic acid, heptadecanoic acid, stearic acid, oleic acid, linoleic acid, α-linolenic acid, arachidic acid and eicosadienoic acid, obviously varied among three species, suggesting each species has its own fatty acid pattern. Principal component analysis and hierarchical clustering analysis according to the absolute and relative contents of fatty acids, showed that 30 tested samples could be clearly differentiated according to the species.

**Conclusions:**

These findings demonstrated that GC-MS-based fatty acid profiling coupled with multivariate statistical analysis provides reliable platform to classify these three *Panax* species, which is helpful for ensuring their safety and efficacy.

## Background

Several plants of the *Panax* species (Family Araliaceae), including *Panax ginseng* C. A. Mey. (Asian ginseng), *Panax quinquefolius* L. (American ginseng) and *Panax notoginseng* (Burk.) F.H. Chen (Notoginseng), have been traditionally used as valuable medicinal herbs since the ancient times in the oriental countries. Current phytochemical and pharmacological studies revealed that *Panax* species contain a variety of bioactive ingredients, including triterpene saponins (ginsenosides), fatty acids, polysaccharides and polyacetylenes [1], and exhibit extensively beneficial effects on immune system, central nervous system and cardiovascular system, cancer and diabetes, etc. [2-4]. The ginsenosides and polysaccharides have been generally considered to be their main bioactive components. In the past years, fatty acids, traditionally viewed as the source of energy, have attracted interest for research and public health, due to their effects on human health and diseases [5]. The unsaturated fatty acids, including monounsaturated fatty acids (MUFA) and polyunsaturated fatty acids (PUFA), are health-promoting and have significant metabolic and cardiovascular benefits [6-8]. There is convincing evidence that the diets rich in α-linolenic acid, a plant-derived omega-3 PUFA, are associated with decreased incidence and severity of several chronic diseases, including hyperlipidemia [9], arrhythmia [10], rheumatoid arthritis [11] and cancer [12,13]. Therefore, fatty acids in *Panax* species might substantially contributed to the whole beneficial effects of herbs, besides ginsenosides and polysaccharides. Up to date, the comparative study on the fatty acids of three main medicinal *Panax* herbs has not been addressed yet.

Although the morphological appearances of *P. ginseng*, *P. quinquefolius* and *P. notoginseng* are similar, their traditional indications are significantly different according to Chinese pharmacological principles [14]. With the increasing market demand and profits temptation, the phenomena that some species are substituted and/or adulterated by other cheaper species appeared due to their different prices [15]. Thus, the authentication of these medicinal herbs is very important for ensuring the safety and efficacy of medication. A few methods, such as liquid chromatography – mass spectrometry (HPLC-MS) [16,17] and liquid chromatography – evaporative light scattering detector (HPLC-ELSD) [18], have been developed to distinguish them according to the diversity of ginsenosides. Very recently, the differences in volatile compositions and plant metabolites were applied to discriminate these three *Panax* herbs using gas chromatography – mass spectrometry (GC–MS) [19] and proton nuclear magnetic resonance spectroscopy (1H NMR) [20], respectively. In the past decade, fatty acid profiling has been extensively used as a sensitive and reproducible biomarker and signature for characterizing microbial communities, such as bacteria and fungi [21-23]. We hypothesized that fatty acid profiling might be developed differences among *Panax* species.

Therefore, the analysis of fatty acids in three *Panax* species is not only beneficial to the elucidation of their pharmacological activities but also important for their authentication. In the present study, the fatty acids of three *Panax* species were characterized and compared using GC-MS followed by multivariate statistical analysis.

## Experimental

### Materials and chemicals

Twenty-one batches of commercial samples, labeled as ginseng (raw *P. ginseng*, G1-G8) and America ginseng (*P. quinquefolius,* AG1-AG13), were purchased from 21 drugstores of mainland China (14) and Macao (7). While, nine bathes of *P. notoginseng* (NG1-NG9) were collected from Yunnan province of China. The botanical origin of materials were identified carefully according to HPLC-ELSD method established previously [18], but sample of AG-3 were identified as *P. ginseng* rather than as *P. quinquefolius*, as claimed by the merchant. All voucher specimens were deposited at 4°C in Institute of Chinese Medical Sciences, University of Macau, Macao, China.

HPLC-grade methanol, *n*-hexane and boron trifluoride (BF3) in methanol (14%) were purchased from Sigma-Aldrich (St. Louis, MO, USA). The GLC-461 reference standards composed of 32 fatty acid methyl esters (FAMEs) and the internal standard, lignoceric acid (C24:0, ≥99.0%), were from Nu-Chek Prep (Elysian, MN, USA). The mixed standards specifically include methyl butyrate, methyl caproate, methyl caprylate, methyl laurate, methyl myristate, methyl myristoleate, methyl pentadecanoate, methyl palmitate, methyl palmitoleate, methyl heptadecanoate, methyl 10-heptadecenoate, methyl stearate, methyl oleate, methyl linoleate, methyl linolenate, methyl 6-9-12-gamma linolenate, methyl arachidate, methyl 11-eicosenoate, methyl 11-14-eicosadienoate, methyl 11-14-17-eicosatrienoate, methyl 8-11-14-homogamma linolenate, methyl arachidonate, methyl eicosapentaenoate, methyl behenate, methyl erucate, methyl docosadienoate, methyl docosapentaenoate, methyl docosahexaenoate, methyl lignocerate and methyl nervonate. Deionized water was purified by a Milli-Q purification system (Millipore, Bedford, MA, USA).

### Sample preparation

The protocol is based on the simplified method of Kang [24,25] with some modifications. Briefly, the air-dried sample was pulverized, 50.0 mg of fine powder was accurately weighted and transferred to glass methylation tube, then mixed with 1.5 mL of hexane, 1.5 mL of 14% BF3/methanol regent and 30 μg lignoceric acid (internal standard). After blanketed with nitrogen, the mixture was heated at 100°C in MK200-2 dry bath incubator (AoSheng, Hangzhou, China) for 1 h and cooled down to room temperature. Methyl esters were extracted in hexane phase after the addition of 1 mL H_2_O and then centrifuged for 5 min at 3000rpm. The upper hexane layer was removed and concentrated under liquid nitrogen gas, and the residue was re-dissolved in 200 μL hexane, subsequently subjected to GC-MS analysis. It must be mentioned that extreme care was taken to avoid mixture exposure to the air at high temperature, in order to prevent PUFA oxidation at all stages of sample preparation.

### GC–MS analysis

GC–MS analysis was conducted on an Agilent 6890 gas chromatography instrument coupled to an Agilent 5973 mass spectrometer and Agilent ChemStation software (Agilent Technologies, Palo Alto, CA). An Omegawax™ 250 fused silica capillary column (30 m × 0.25 mm i.d., 0.25 μm film thickness, Supelco, Belletonte, PA) was used for separation. High purity helium was used as carrier gas with the flow rate of 1.5 mL/min. The optimized column temperature program was as follows: initial temperature set at 180°C and held for 3 min; ramped to 240°C at 2°C /min, held at 240°C for 7 min. Split injection (10 μL) with a split ratio of 1:15 was used and temperature of injector was set at 250°C. The spectrometer was operated in electron-impact (EI) mode with ionization energy of 70 eV, scan range was 35–550 atomic mass unit (amu) between 3 min to 40 min and scan rate was 0.34 s per scan. The quadrupole and ionization source temperature were 150°C and 280°C, respectively.

### Identification and quantification of fatty acids

Fatty acids were identified as their methyl esters by three means: (i) by searching potential structures from NIST MS Search 2.0 database, (ii) by comparing retention time of peaks with those of reference compounds eluted under the same chromatographic conditions, (iii) by comparing their mass spectra with those of authentic standards. The absolute concentrations of fatty acids were quantified by comparing their peak areas to that of internal standard on the GC-MS chromatogram. The relative content of each fatty acid in these herbs was also calculated by normalization of the peak areas as the percentages of total fatty acids.

### Method validation

Intra-day variation was chosen to determine the precision of GC-MS method. The mixed standards were analyzed for six times within 1 day. In order to examine the inherent stability characteristics of methylated fatty acids, freshly prepared *P. ginseng* sample (G-8) was analyzed at different time intervals of 0h, 1h, 2h, 3h, 4h and 6h. In addition, to test repeatability of methylation, sample of *P. notoginseng* (**NG-3)** was divided into six and parallelly derivatizated under the methylation conditions, and then analyzed by GC-MS as mentioned above. Their variations were expressed by relative standard deviations (RSD).

### Statistic analysis

The absolute and relative contents of fatty acids were expressed as Mean ± SD. Analysis of variance (ANOVA) was performed to assess statistical difference in the content of each fatty acid from three *Panax* species using SPSS version 19.0 software (SPSS, Inc., Chicago, IL, USA). To evaluate correlation of 30 tested samples, hierarchical cluster analysis (HCA) was used to generate the dendrogram using SPSS according to fatty acids characteristics from GC-MS profiles. A method named as Ward, a very efficient method for analysis of variance between clusters, was selected as measurement. In addition, the absolute and relative contents of fatty acids were respectively imported into SIMCA-P version 13.0 (Umetrics, Umeå, Sweden) for unsupervised principal component analysis (PCA). PCA was carried out to obtain an overview of the variations among groups using Pareto scaling which is commonly used in metabolomic studies.

## Results and discussions

### Method validation

The robustness or ruggedness of analytical method, including instrumental analysis and sample preparation, should be evaluated to guarantee statistical difference is not derived from analytical drift in a chemometric study. The mixed standards were measured in succession for six times and 11 investigated fatty acids were selected to monitor the instrumental drift. As results, the peak area variations of fatty acids were less than 5% (data not shown), suggesting good instrumental performance during the whole analytical run. Several fatty acids containing unsaturated double bond, particularly PUFA, could be facilely oxidized after long-term exposure to the air. Therefore, the stability characteristics of methylated fatty acids were tested. As shown in Table 1, FAMEs derived from test sample (G-8) were stable for at least 6 hours at ambient room temperature with overall variation of 0.34%-9.02%. In addition, the repeatability of methylation detected in test sample (NG-3) was less than 5.31%. The coherence of retention time was important for the subsequent peak identification and peak picking. The retention time of each FAME was found to be consistent (less than 0.02 min) during the whole analysis.

**Table 1:** Stability and reproducibility data of 11 investigated fatty acids

**Peak No.**	**Stability test (G-8)**			**Reproducibility test (NG-3)**		
	**RT (min)**	**Relative content (%)**	**RSD (%)**	**RT (min)**	**Relative content (%)**	**RSD (%)**
**1**	3.98 ± 0.01	0.25 ± 0.02	6.40	3.98 ± 0.01	0.48 ± 0.03	5.31
**2**	5.14 ± 0.01	0.92 ± 0.02	2.21	5.13 ± 0.01	0.46 ± 0.02	3.94
**3**	6.67 ± 0.01	25.82 ± 0.49	1.90	6.65 ± 0.00	30.12 ± 0.27	0.91
**4**	7.04 ± 0.01	1.82 ± 0.07	4.09	−	−	−
**5**	8.52 ± 0.01	0.67 ± 0.01	1.45	8.51 ± 0.00	0.56 ± 0.02	4.05
**6**	10.76 ± 0.01	2.04 ± 0.05	2.27	10.74 ± 0.01	2.42 ± 0.05	2.00
**7**	11.28 ± 0.01	7.68 ± 0.10	1.32	11.26 ± 0.00	10.59 ± 0.21	1.94
**8**	12.54 ± 0.02	55.66 ± 0.19	0.34	12.47 ± 0.00	50.12 ± 0.23	0.47
**9**	14.22 ± 0.01	2.96 ± 0.05	1.71	14.20 ± 0.00	3.81 ± 0.08	2.21
**10**	16.13 ± 0.01	1.20 ± 0.11	9.02	16.10 ± 0.01	1.43 ± 0.02	1.67
**11**	18.16 ± 0.01	0.97 ± 0.07	6.96	−	−	−

Data represents mean ± SD. The samples of *P. ginseng* (G-8) and *P. notoginseng* (NG-3) were employed to stability and reproducibility tests, respectively (n = 6). RT, retention time.

### Fatty acid composition of three *Panax* species

**Figure 1:**
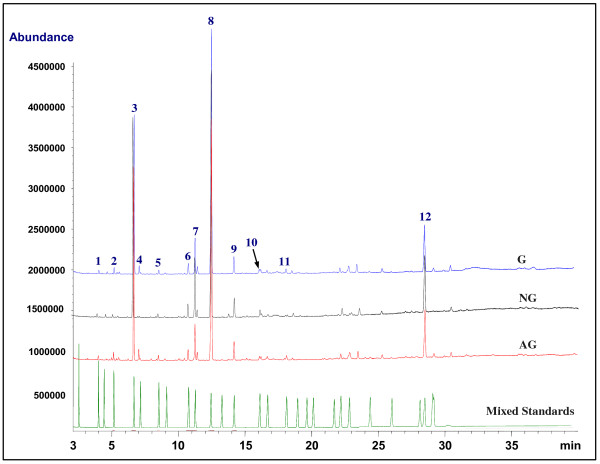
**Typical GC chromatograms of *P. ginseng *(G), *P. notoginseng *(NG), *P. quinquefolius *(AG) and mixed standards determined by GC-MS.** Fatty acid represents as the corresponding methyl ester. **1**, myristic acid; **2**, pentadecanoic acid; **3**, palmitic acid; **4**, palmitoleic acid; **5**, heptadecanoic acid; **6**, stearic acid; **7**, oleic acid; **8**, linoleic acid; **9**, α-linolenic acid; **10**, arachidic acid; **11**, eicosadienoic acid; **12**, lignoceric acid (Internal standard). The mixed standards contain 32 fatty acid methyl esters as described in section of Chemicals.

Typical GC chromatograms of the mixed standards containing 32 FAMEs and three *Panax* species were shown in Figure 1. Under the GC-MS conditions mentioned above, the peaks corresponding to the investigated fatty acids were well separated on an Omegawax 250 column within 35 min. Eleven fatty acids were found and characterized in *P. ginseng* and *P. quinquefolius*, mainly by comparison of their retention time and mass spectra with those obtained from reference compounds under the same chromatographic conditions. Among these fatty acids, palmitoleic acid (C16:1 n-9) and eicosadienoic acid (C20:2 n-6) were not detected in all samples of *P. notoginseng*. However, it was still very hard to discriminate three different *Panax* species by visual observation of the fatty acid profiles detected by GC-MS, as the major components across samples were very similar, especially in *P. ginseng* and *P. quinquefolius*. By comparing their peak areas to that of internal standard and normalizing the peak areas as percentage, the absolute (μg/g) and relative content (%) of eleven fatty acids in three *Panax* species were calculated, respectively, as summarized in Table 2. Linoleic acid (C18:2 n-6), palmitic acid (C16:0) and oleic acid (C18:1 n-9) were main fatty acids in three *Panax* species. *P. ginseng* showed the highest absolute content of total fatty acids. Furthermore, the content of each fatty acid, saturated fatty acids (SFA), MUFA, PUFA and the ratio of omega-6/omega-3, obviously varied in three species, which suggested that each species had its own fatty acid pattern.

**Table 2:** **The absolute and relative contents of fatty acids in *P. ginseng *(G, n = 8), *P. notoginseng *(NG, n = 9) and *P. quinquefolius *(AG, n = 12)**

**Fatty acids**			**Absolute content (μg/g)**			**Relative content (%)**		
**No.**	**Common name**	**Symbol**	**G**	**NG**	**AG**	**G**	**NG**	**AG**
**1**	Myristic acid	C14:0	0.16 ± 0.03^a^	0.20 ± 0.05^a^	0.28 ± 0.08^b^	0.41 ± 0.26^a^	0.54 ± 0.14^a^	0.70 ± 0.14^b^
**2**	Pentadecanoic acid	C15:0	0.47 ± 0.05^a^	0.15 ± 0.01^b^	0.34 ± 0.04^c^	0.89 ± 0.08^a^	0.42 ± 0.06^b^	0.86 ± 0.14^a^
**3**	Palmitic acid	C16:0	13.93 ± 1.52^a^	10.49 ± 1.22^b^	14.69 ± 1.04^a^	26.31 ± 0.92^a^	29.39 ± 1.84^b^	36.92 ± 4.76^c^
**4**	Palmitoleic acid	C16:1 (n-9)	0.71 ± 0.17^a^	−	0.18 ± 0.07^b^	1.33 ± 0.27^a^	−	0.45 ± 0.17^b^
**5**	Heptadecanoic acid	C17:0	0.36 ± 0.04^a^	0.21 ± 0.03^b^	0.51 ± 0.07^c^	0.69 ± 0.09^a^	0.62 ± 0.12^a^	1.27 ± 0.19^b^
**6**	Stearic acid	C18:0	1.41 ± 1.02	1.05 ± 0.55	1.50 ± 0.20	2.63 ± 1.71^ab^	2.84 ± 1.09^a^	3.78 ± 0.80^b^
**7**	Oleic acid	C18:1 (n-9)	3.88 ± 0.55^a^	3.96 ± 1.22^ab^	2.98 ± 0.61^b^	7.37 ± 0.97^a^	11.03 ± 2.81^b^	7.38 ± 1.23^a^
**8**	Linoleic acid	C18:2 (n-6)	28.22 ± 2.3^a^	17.98 ± 2.41^b^	17.47 ± 4.34^b^	53.51 ± 2.93^a^	50.25 ± 3.94^a^	42.58 ± 5.28^b^
**9**	α-linolenic acid	C18:3 (n-3)	1.71 ± 0.26^a^	1.25 ± 0.22^b^	1.53 ± 0.45^ab^	3.24 ± 0.45	3.49 ± 0.53	3.72 ± 0.73
**10**	Arachidic acid	C20:0	0.45 ± 0.09^a^	0.50 ± 0.20^a^	0.70 ± 0.20^b^	0.85 ± 0.16^a^	1.40 ± 0.55^b^	1.78 ± 0.68^b^
**11**	Eicosadienoic acid	C20:2 (n-6)	0.52 ± 0.07 ^a^	−	0.24 ± 0.05^b^	0.98 ± 0.14^a^	−	0.58 ± 0.10^b^
Total FA			51.84 ± 4.18^a^	35.80 ± 4.31^b^	40.41 ± 5.49^b^	100.00	100.00	100.00
SFA			16.78 ± 2.18^a^	12.61 ± 1.84^b^	18.02 ± 0.95^a^	31.77 ± 3.22^a^	35.22 ± 2.80^b^	45.31 ± 5.63^c^
MUFA			4.59 ± 0.67^a^	3.96 ± 1.22^ab^	3.16 ± 0.64^b^	8.70 ± 1.24^ab^	11.02 ± 2.81^a^	7.82 ± 1.26^b^
PUFA			30.45 ± 2.33^a^	19.23 ± 2.57^b^	19.23 ± 4.79^b^	57.73 ± 3.51^a^	53.74 + 4.26^a^	46.87 + 5.92^b^
n-6/n-3			17.17 ± 2.7^a^	14.65 ± 1.88^a^	11.85 ± 1.48^b^	17.16 ± 2.70^a^	14.65 ± 1.88^a^	11.86 ± 1.48^b^

Data represents mean ± SD. Averages followed by different letters are significantly different (*P* < 0.05). FA, fatty acid; SFA, saturated fatty acids; MUFA, monounsaturated fatty acids; PUFA, polyunsaturated fatty acids; n-6/n-3: ratio of omega-6 to omega-3 fatty acids.

### Comparison of fatty acids in three *Panax* species

**Figure 2:**
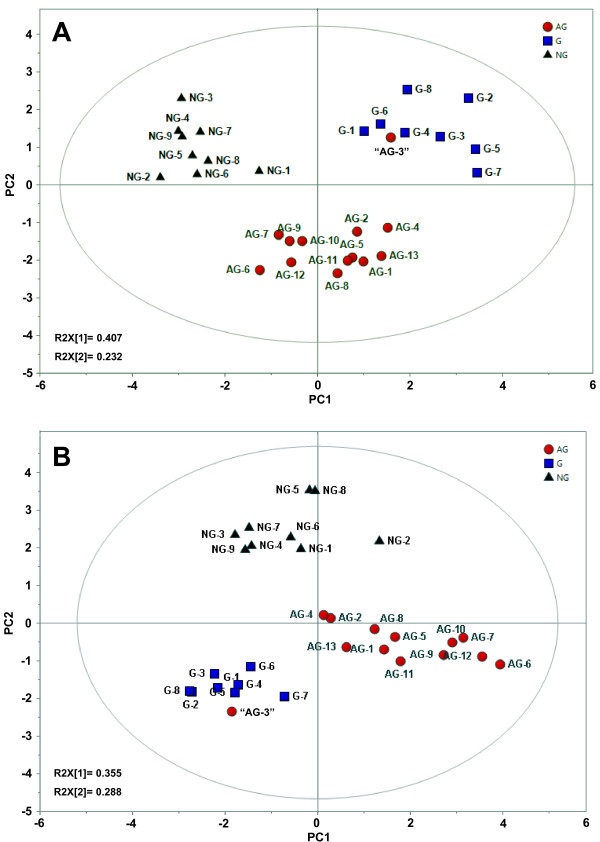
**PCA score plots based on absolute content (A) and relative content (B) of fatty acids in *P. ginseng *(G, n = 8), *P. notoginseng *(NG, n = 9) and *P. quinquefolius *(AG, n = 13).** Sample of “AG-3”, claimed to be *P. quinquefolius* by vendor, was actually identified as *P. ginseng.*

With the data sets of absolute and relative contents, PCA, an unsupervised multivariate statistical method for pattern recognition, was applied to distinguish three *Panax* species according to their difference in fatty acids. After unit variance (UV) scaling and mean-centering, all data were displayed as scores and loadings in a coordinate system of principal components resulting from data dimensionality reduction. As results, two-dimensional PCA score plots according to absolute content of fatty acids showed clear discrimination of three *Panax* species, explaining 63.9% of total variance (Figure 2A). *P. quinquefolius* was preferentially separated from *P. notoginseng* and *P. ginseng* by principal component 2 (PC2, 40.7%), while *P. notoginseng* and *P. ginseng* could be distinguished each other by PC1 (23.2%). In score plots based on relative content, although, each *Pa*nax species was presented in distinctly different region, these three herbs could not be much clearly discriminated by PC1 (35.5%) and PC2 (28.8%) (Figure 2B). It is plausible that, comparing to absolute content, relative content of several fatty acids might narrow their difference among different species by normalizing peak areas as percentages of total fatty acids. For example, significant difference in the absolute content, but not relative content, of α-linolenic acid, an important plant-derived omega-3 fatty acid, was found among three species (Table 2). It is interesting that the sample of “AG-3” claimed to be *P. quinquefolius* by vendor was actually presented in the cluster regarding to *P. ginseng*, in accordance with the identification of materials above. The loading plots displayed the correlation structure of the variables, in the present study, and showed which fatty acids describe the similarity and dissimilarity observed in three *Panax* species. The PCA loading plots based on absolute contents indicated that palmitic acid (C16:0) (**3**), palmitoleic acid (C16:1) (**4**), heptadecanoic acid (C17:0) (**5**) and linoleic acid C18:2 (n-6) (**8**), collectively induced the crucial distinction among *Panax* species. Besides, C16:1 and C17:0 also played a prominent role in distinguishing the relative contents of fatty acids in these herbs. Thus, PCA was applied to successfully differentiate *Panax* species.

**Figure 3:**
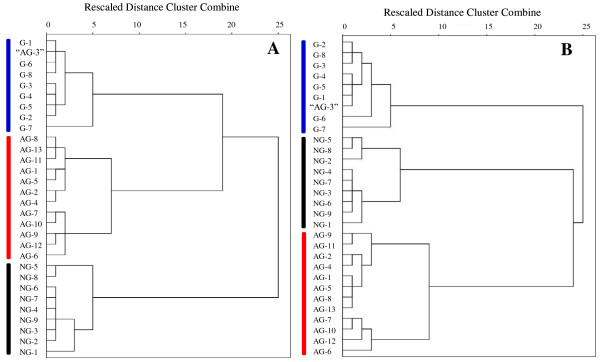
**Dendrograms of HCA resulting from absolute content (A) and relative content (B) of 11 fatty acids in 30 tested *Panax *samples.** Sample of “AG-3”, claimed to be *P. quinquefolius* by vendor, was actually identified as *P. ginseng.*

**Figure 4:**
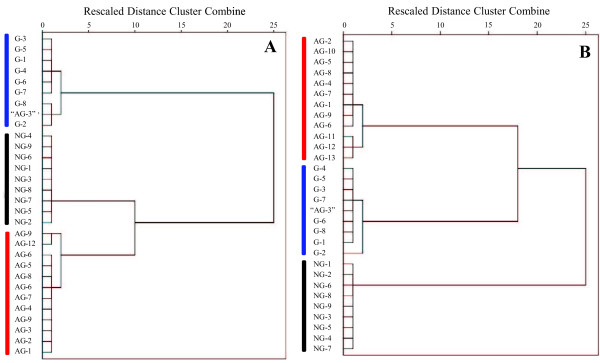
**Dendrograms of HCA resulting from absolute content (A) and relative content (B) of palmitoleic acid (C16:1) and heptadecanoic acid (C17:0) in 30 tested *Panax *samples.** Sample of “AG-3”, claimed to be *P. quinquefolius* by vendor, was actually identified as *P. ginseng*.

In addition, respectively based on absolute and relative contents of 11 fatty acids, HCA of tested 30 samples was performed using a Ward method to visualize the differences and/or similarities among samples through linkage distances. The HCA dendrograms, derived from both absolute and relative contents, showed that the samples derived from three species could be divided into three main clusters (Figure 3), each species corresponding to a cluster. In accordance with the PCA results, the sample of “AG-3” corresponded to the *P. ginseng* in HCA, rather than *P. quinquefolius.* Using the content of C16:1 and C17:0, selected fatty acids in PCA loading plots, HCA (Figure 4) of tested 30 samples were also performed as mentioned above. The results were similar to that derived from the content of 11 fatty acids. Therefore, the characteristics of fatty acids, especially C16:1 and C17:0, might be used as markers for discrimination of these three *Panax* species. These results suggested that by using absolute and relative contents of fatty acids, the *Panax* species could be discriminated using GC-MS analysis and multivariate statistical analysis, such as PCA and HCA.

During the last decades, fatty acid profiles have been widely used to characterize microbial community [26]. GC analysis of fatty acids, in particular, derived from phospholipid, has proven to be highly applicable method for the bacteria species identification [27-30]. Phospholipids naturally presented in fresh medicinal materials, but might be destructed and decomposed during traditional processing procedure [31-33]. Therefore, the total fatty acid derived from all types of lipids, such as free fatty acids, phospholipids and triglycerides, in herbal tissue, was used to characterize the *Panax* species in this study.

## Conclusions

In the present study, we demonstrated that using GC-MS followed with multivariate statistical analysis, fatty acid profiling provides reliable discrimination among *P. ginseng*, *P. notoginseng* and *P. quinquefolius.* Based on absolute and relative content of fatty acids, the authentication of the three *Panax* species could be well performed, which is helpful for ensuring their safety and efficacy.

## Abbreviations

ANOVA: Analysis of variance; AG: American Ginseng (*Panax quinquefolius)* ; BF3: Boron trifluoride; EI: Electron-impact; FAMEs: Fatty acid methyl esters; G: Ginseng (*Panax ginseng*); GC-MS: Gas chromatography – mass spectrometry; HCA: Hierarchical cluster analysis; 1H NMR: Proton nuclear magnetic resonance spectroscopy; HPLC-ELSD: Liquid chromatography – evaporative light scattering detector; HPLC-MS: Liquid chromatography – mass spectrometry; MUFA: Monounsaturated fatty acids; NG: *Panax notoginseng* ; PCA: Principal component analysis; PUFA: Polyunsaturated fatty acids; RSD: Relative standard deviations; UV: Unit variance.

## Competing interests

The authors declare that they have no competing interests.

## Authors’ contributions

JBW initiated and all authors designed the study. The sample preparation and GC-MS method development were carried out by XJZ who drafted the manuscript. All authors contributed to data analysis and manuscript finalization. All authors read and approved the final manuscript.
